# Cancer Immunotherapy Using Chimeric Antigen Receptor Expressing T-Cells: Present and Future Needs of Clinical Cancer Centers

**DOI:** 10.3389/fimmu.2020.565236

**Published:** 2020-10-30

**Authors:** Manuel Gotti, Irene Defrancesco, Mario D’Angelo, Sabrina Basso, Luca Crotto, Alfredo Marinelli, Cristina Maccalli, Vincenzo Iaconianni

**Affiliations:** ^1^Division of Hematology, Fondazione Istituto di Ricovero e Cura a Carattere Scientifico (IRCCS) Policlinico San Matteo, Pavia, Italy; ^2^Department of Molecular Medicine, University of Pavia, Pavia, Italy; ^3^Department of Onco-hematology, Cell and Gene Therapy, Ospedale Pediatrico Bambino Gesù IRCCS, Roma, Italy; ^4^Pediatric Hematology/Oncology Unit, Fondazione IRCCS Policlinico San Matteo, Pavia, Italy; ^5^Cell Factory, Fondazione IRCCS Policlinico San Matteo, Pavia, Italy; ^6^Candiolo Cancer Institute, FPO–IRCCS, Candiolo, Italy; ^7^Operative Unit (OU) Neuroncology, University Federico II, Napoli, Italy; ^8^IRCCS Neuromed Istituto Neurologico Mediterraneo Pozzilli (INM), Pozzilli, Italy; ^9^Laboratory of Immune and Biological Therapy, Research Department, Sidra Medicine, Doha, Qatar; ^10^ ICMED, Sesto San Giovanni, Italy

**Keywords:** CAR-T cells, CAR-T process, CAR-T Unit, CAR-T Specialist, JACIE, GMP, ATMP

## Abstract

Chimeric Antigen Receptor-T cells (CAR-T) are considered novel biological agents, designed to selectively attack cancer cells expressing specific antigens, with demonstrated clinical activity in patients affected with relapsed/refractory B-cell malignancies. In consideration of their complexity, the use of CAR-T requires dedicated clinical setting and health care practitioners with expertise in the selection, treatment, and management of toxicities and side effects. Such issue appears particularly important when contextualized in the rapid progress of CAR-T cell treatment, translating into a constant need of updating and evolution. Moreover, the clinical grade manufacturing of CAR-T cells is complex and implies articulated regulatory and organizational aspects. The main goal of this review is to summarize and provide an accurate analysis of the clinical, logistic, and regulatory requirements of CAR-T cell centers. Finally, we describe a new occupational figure called “CAR-T specialist” devoted to the establishment and coordination of the required facilities and regulatory landscape in the context of cancer centers.

## Introduction

The demonstration that the immune system can control tumor growth has been provided for the first time by Thomas and Burnet (1, 2). This evidence has been confirmed after several decades, demonstrating the prognostic role of immune cells infiltrating the tumor lesions (3–10). The crescent knowledge of cancer immunology and immunotherapy has allowed the development of novel biological agents that showed unprecedented clinical results (11). These results contributed to turn into reality the paradigm that patient’s immune system represents effective “living drugs” against cancer cells. Among these, adoptive cell therapy (ACT) that implies the isolation and expansion *ex vivo* of tumor antigen-specific T lymphocytes and their re-infusion in patients has shown promising clinical activity improving the overall survival of cancer patients (12). T-lymphocytes engineered with Chimeric Antigen Receptors (CARs), that consist of an antibody-derived domain for antigen recognition linked to T-cell signaling molecules, can recognize in a MHC-independent manner tumor antigens expressed on tumor cell surface (13–17). These biological drugs are based on the engineering of T-lymphocytes, isolated from patient’s or, less frequently, from donor’s peripheral blood, with either gamma-retroviral (RV) or lentiviral (LV) vectors encoding CARs linked to co-stimulatory molecules (either CD28 or 4-1BB linked to zeta-chain) (18–20). The development of anti-CD19 CAR-T cells and their application in several clinical trials showed durable clinical responses in both adult and pediatric cancer patients with disease relapse or refractory to other therapeutic interventions. Complete responses (up to 70–80%) and significant improvement of overall survival (OS) were documented in patients with either acute lymphoblastic leukemia (ALL) (21, 22) or high grade non-Hodgkin lymphomas (NHLs), including diffuse large B-cell lymphoma (DLBCL) (23, 24) and mantle cell lymphoma (ML) (25). These clinical trials led to the accelerated approval by the U.S. Food and Drug Administration (FDA) and the European Medicines Agency (EMA) of two CD19-CAR-T cell medicinal drug products, tisagenlecleucel (Kymriah) and axicabtagene ciloleucel (Yescarta). For the first time, ACT products entered commercial production by few pharmaceutical companies. Nevertheless, the clinical grade manufacturing is complex and with relatively long timeline for cell production, requiring the availability of patients’ or donors’ peripheral blood mononuclear cells (PBMCs) to be shipped to good manufacturing practice (GMP) facilities, if not locally available, and the return to clinical sites for infusion into patients. One of the challenging factors of CAR-T cells is represented by the short-term associated toxicities arising in patients immediately after infusion. Cytokine release syndrome (CRS) represents the most common side effects of this type of therapy with a range of incidence among the different clinical studies of 50–90% (26). The mechanistic hypothesis behind CRS still needs to be fully dissected; however, it is a life-threatening situation that requires timely and effective interventions (27). Other common side effects observed after CAR-T cell therapy for B-cell malignancies involve neurotoxicity, B-cell aplasia and hypogammaglobulinemia (26). CAR-T cells have been also investigated for the treatment of solid tumors with limited clinical responses, thus suggesting that this therapeutic strategy might be promising, but still needs optimization. One of the principal limitations is represented by the choice of target antigens; the majority of solid tumors have epithelial origins with many of tumor-associated antigens (TAAs) being shared with normal tissues (28, 29). This translates into high risk of inducing off-tumor toxicities following the infusion of CAR-T cells specific for these TAAs. In addition, the homing of CAR-T cells to tumor site for solid tumors showed low efficiency due to the complexity of the tumor microenvironment (TME) (30). Multiple studies are aimed at addressing the choice of target molecules, such as IL-13alpha2, B7-H3, CSPG4, CD44v6, MUC1, Mesothelin, EGFRvIII, Her-2, GD2 *etc*., allowing to specifically redirect the CAR-T cells to tumors with selected histological origins (31–37). A remarkable number of clinical trials are ongoing worldwide to assess the clinical activity of these immunobiological drugs against solid tumors and the optimal clinical setting for cancer patients (28, 29, 35).

The organizational and regulatory aspects of CAR-T cell clinical center represent novel and challenging topic within a rapidly evolving field. These involve different clinical expertise and departments, regulatory and quality management, laboratory dedicated to the tissue collection, processing and storage, and the accurate and timely coordination of logistics. The complexity of this organization requires personnel with specific background and continuous interactions and coordination among them. This manuscript aims at addressing the clinical, regulatory, and organizational requirements of clinical centers, including hematological and oncological departments, dedicated to CAR-T cell therapies called CAR-T Unit. In this review a model of CAR-T Unit is proposed, as well as the need of a new figure called “CAR-T specialist”. The connection between the CAR-T Unit and the involved facilities, either within the clinical centers (*e.g.*, clinical unit, apheresis unit, processing unit, pharmacy, intensive care unit) or external (*e.g.*, pharma companies, clinical research organizations) is also described.

## The Multifaced Evolution of CAR-T Cells

In order to achieve clinical efficacy, the CAR-T cell therapy should have four features: i) high effect or activity, even in the immunosuppressive tumor environment; ii) high proliferation capacity, even after multiple encountering with cancer cells; iii) long-term persistence; iiii) absence or low degree of toxicity after cell infusion.

Despite the documented clinical success of anti-CD19 CAR-T cells, the broad clinical usage is still limited due to several factors, such as the non-optimal persistence, the low efficacy in solid tumors, the safety profile and the high costs associated with the complex manufacturing. Although anti-CD19 CAR-T cells can lead to complete response rates for B-cell malignancies, a consistent proportion of patients relapse through various resistance mechanisms, including loss of expression of the target antigen (CD19-negative recurrences) (38). To overcome this drawback and reduce the risk of disease recurrence, T cells have been engineered with multiple CARs to target simultaneously two tumor-associated antigens (bispecific CAR-T cells) (39–41). This strategy may allow decreasing the toxicity while augmenting the tumor specificity of CAR-T cells. To improve their anti-tumor efficacy, they have been implemented with molecular features that can target the tumor microenvironment (TME). These are represented by the fourth generation “armored” CAR-T cells that include T cells redirected for Universal Cytokine-mediated Killing cells (TRUCK) through the incorporation of genes encoding for cytotoxic cytokines (42). Moreover, allogeneic or “universal” CAR-T cells are produced from T cells isolated from healthy donors, representing “off the shelf” engineered T cells. This strategy allows the viability of CAR-T cells for a high number of patients and the decrease of the costs regarding clinical grade production (43). Great emphasis is also placed in the development of CAR-T cells for the treatment of solid malignancies; to date results showed limited clinical efficacy (44–48). The challenges associated with the effectiveness of CAR-T cells in this setting are: i) the selection of target antigens, specifically expressed by tumor and not by normal cells; ii) the homing of CAR-T cells and their interaction with TME and the vascularization; iii) the production of immunosuppressive molecules; iiii) the high metabolic rate of both tumor cells and TME (49). To date in fact, a candidate target antigen expressed by solid tumors that is comparable to CD19, a lineage antigen over-expressed on B-cell malignancies which counterpart expression on normal cells limited to B cells is not available. Due to the high heterogeneity of cells within solid tumors, the “armored” CAR-T cells allow promotion of a universal cytokine-mediated activation, targeting even those cancer cells that would be invisible to CAR-T cells.

Notably, the ability to penetrate the TME, the biodistribution, survival, and proliferative features of CAR-T cells need to be considered for the efficient redirection of these cells to tumor cells. The antigen expression and localization on tumor cells is one of the limiting factors for the anti-tumor activity of CAR-T cells; nevertheless, the immunomodulatory activity of the TME can influence the fate of immune responses and the outcome. The combination of CAR-T cells with immune checkpoint blockade or other immunomodulatory agents represents a promising therapeutic intervention that is currently under evaluation (37, 50). The *in situ* administration of CAR-T cells could overcome their limited migration to solid tumors (51). In neuro-oncology this strategy can be applicable through intralesional and intraventricular administrations of the cells since the peculiarity of the cerebral circulatory system allows the spreading of the CAR-T cells (51).

## CAR-T Cell Program

CAR-T cell therapies have been classified in the regulatory category of Advanced Therapy Medicinal Products (ATMPs) under the definition of a gene therapy medicinal product, although they represent cellular therapies. To assure the reliability and safety of this type of therapy, it is important to define the optimal conditions of administration to patients. Therefore, an organizational structure with accreditation path needs to be identified. In 2009, a specialized committee was created by the EMA, the Committee for Advanced Therapies (CAT) (Regulation (EC) No. 1394/2007of the European Parliament and of the Council of November 13, 2007), aimed at providing opinion on the quality, safety, and efficacy of studies utilizing CAR-T cells. Details of the structure, experience, and regulatory requirements of clinical centers where CAR-T cell therapy can be administered are discussed in the text below. The initial indication has been established for the hematology field, for which approved medicinal products are available. To guarantee the safety of patients, the requirements for CAR-T cells centers have been identified as similar to those for early-phase clinical trials for novel immune therapies, with the designation of clinical centers with documented experience in allogeneic hematopoietic stem cell transplantation (HSCT) program, from alternative donors and sources, as reference for this type of therapy. This implies the availability of on-site clinical hematology unit, with intensive care unit, neurological department, emergency department, pharmacy, and transfusion center.

Long-term trained and experienced multi-disciplinary teams working together to care for intensively treated cancer patients are mandatory. Nevertheless, regulatory and logistic landscape of this cell-based therapy displays high complexities. Therefore, an accurate planning of infrastructures and identification of specific guidelines are needed to develop cell-therapy programs. The details of organizational and regulatory requirements are explained in the following paragraphs and sub-paragraphs. Of note, the administration of CAR-T cells to patients with either hematological or solid tumors requires joint transplant programs that include different figures, organization charts, and quality systems. Given the complexity of this scenario, the so called “CAR-T specialist” has great expertise in regulatory aspects and therefore has a relevant role in identifying, controlling, and implementing the specific requirements that a “CAR-T Unit” should obtain.

## Regulatory Guidelines

In order to analyze the regulatory aspects of CAR-T cell therapy, the following premises are of relevant value: i) CAR-T cells have to be considered “Drug Products”; ii) prior to their production, a deep knowledge of the legislation concerning the whole process (from lymphocytes collection to cells infusion). Lymphocytes are both blood components and cells; therefore their manipulation is controlled by legislation on blood components, tissue, and cells.

Taken into consideration the above points, the process of CAR-T cell generation requires the compliance with the regulation summarized in **Table 1**.

**Table 1:** Regulatory entities for the production of CAR-T cells.

	Tissueregulation	Bloodregulation	ATMP	JACIEStandards	GMPregulation
Collection of tissue and cells	X	X		X	
Production			X		X
CAR-T Infusion			X	X	

### Tissue Regulation

Lymphocytes are cells and are regulated by the European guidelines for tissue and cells. The legal framework defining the safety and quality standards for tissues and cells is based on the *Directive 2004/23/EC*, also referred to as the “European Tissues and Cells Directive”, adopted in 2004 by the European Parliament and Council. It comprises the process for transplant, including donation, procurement, testing, processing, preservation, storage, and delivery. The *Directive 2004/23/EC* has been implemented by the following:

- *Commission Directive2006/17/EC* providing technical requirements for the donation, procurement and testing of human tissues and cells.
- *Commission Directive2006/86/EC* addressing the traceability, notification of severe adverse reactions and events, coding, processing, preservation, storage, and delivery of human tissues and cells to facilities and clinical centers.
- *Commission Directive 2015/565* amending the Directive 2006/86/EC regarding the coding of human tissues and cells.
- *Commission Directive 2015/566* implementing the Directive 2004/23/EC with the procedures for verifying the equivalent standards of quality and safety of imported tissues and cells.
- *Commission Decisions 2010/453/EC* and Commission Directive 2012/39/EU, as well as Commission Decision C(2015)4460

The *Directive 2004/23/EC* specifies that (introduction, point 7):

“Tissues and cells intended to be used for industrially manufactured products, including medical devices, should be covered by this Directive only as far as donation, procurement and testing are concerned, where the processing, preservation, storage and distribution are regulated by other Community legislation. The further manufacturing steps are covered by Directive 2001/83/EC of the European Parliament and of the Council of 6^th^ November 2001 on the Community code relating to medicinal products for human use(4)”.

Thus, only donation and donor selection/testing are regulated by the Directive 2004/23/EC, in particular: i) criteria for donor selection and evaluation, as the document defines how to manage the inform consent of donors and the procedures for data protection and confidentiality; ii) the procurement requirements are illustrated in chapter 15 (*Directive 2004/23/CE*). *“The activities related to tissue procurement shall be carried out in such a way as to ensure that donor evaluation and selection is carried out in accordance with the requirements referred to in Article 28(d) and (e) and that the tissues and cells are collected, packaged and transported in accordance with the requirements referred to in Article 28(f)*.Authorization

An important point of the Directive 2004/23/CE is paragraph 6:

1. “Member States shall ensure that all tissue establishments where activities of testing, processing, preservation, storage or distribution of human tissues and cells intended for human applications are undertaken have been accredited, designated, authorized or licensed by a competent authority for the purpose of those activities.
2. The competent authority or authorities, verified that the tissue establishment complies with the requirements referred to in Article 28(a), shall accredit, designate, authorize or license the tissue establishment and indicate which activities it may undertake, and which conditions apply. It or they shall authorize the tissue and cell preparation processes which the tissue establishment may carry out in accordance with the requirements referred to in Article 28(g). Agreements between tissue establishments and third parties, as referred to in Article 24, shall be examined within the framework of this procedure.”

These guidelines identify that only accredited, designed, authorized or licensed institution can manipulate tissues for cell therapy. Each State Member identifies the criteria for the accreditation, and variability takes place in terms of the authorization criteria applied among different state members in EU. Once tissue isolation has been licensed, accredited, or authorized, it will be registered on the dedicated EU website.

### Regulation for Blood Collection

This topic has multiple guidelines according different regions or countries. In some cases (*i.e.* Italy), this activity is under the responsibility of Transfusion Center in terms of collection of peripheral blood-derived cells. National regulatory agencies provide local procedures and guidelines. This introduces further complexity in the regulatory field of CAR-T cell therapy.

### Joint Accreditation Committee ISCT-Europe (JACIE) Standard Requirements and Accreditation

CAR-T cell production and clinical management have peculiar characteristics that can be associated with HSCT; therefore in most of the Countries in EU the Regulatory Agencies have identified that their administration should occur in a Bone Marrow transplant Unit. This activity is regulated by the Foundation for the Accreditation of Cellular Therapy-Joint Accreditation Committee of the International Society for Cellular Therapy (ISCT) and the European Group for Blood and BMT (FACT-JACIE). The European Society of Blood and Marrow Transplantation (EBMT) implemented the FACT-JACIE international standards for HSCT programs including specific requirements for CAR-T cell preparation and treatment, intended as guidelines for the usage of this novel therapeutic approach (“*The Joint Accreditation Committee ISCT-Europe & EBMT (JACIE) is Europe’s only official accreditation body in the field of hematopoietic stem cell transplantation (HSCT) and cellular therapy. It promotes high-quality patient care and medical and laboratory practice through a professional-driven and voluntary accreditation scheme*”. JACIE has developed quality standards to evaluate the quality level of the transplant programs in all the phases, including the collection of stem cells, the activities of cell processing, and the infusion in patients. The latest version of the JACIE Standard included the accreditation for the administration to patients of Immune Effector Cells (IEC), including CAR-T cells. It is important to note that the JACIE standard is limited to the qualification of centers dedicated to the *stem cell transplant programs* and for this reason only CAR-T products connected to the stem cell unit and utilized for the treatment of hematological malignancies are included. CAR-T cell products for the treatment of solid tumors are not included, and this can generate controversies in the organization of clinical centers for the CAR-T cell therapy of this type of tumors. According to the JACIE standards, the requirements to apply for the CAR-T products are the following:

- Clinical requirements;
- Collection requirements;
- Processing requirements.

The FACT-JACIE standard is a voluntary standard of excellence and aimed at accrediting and qualifying stem cell transplant programs; therefore institutionally it does not cover the activities for solid tumors. However, an HPC-A transplant program includes several units: the clinical unit that performs the transplant, the aphaeretic collection center, and the cell manipulation laboratory that processes the products. The clinical unit takes care of the therapeutic aspects while the aphaeretic collection unit and the cell manipulation laboratory are not strictly dependent on the clinical hematological activity. The activities of the aphaeretic collection unit and the cell manipulation laboratory are focused on the donor and the cellular product; therefore, these activities can be performed also for oncological clinical units for the CAR-T cell therapy of patients with solid tumors. The requirements of the FACT-JACIE standards related to apheresis and cell manipulation are relevant for the cell product collection and manipulation regardless the type and origin of tumor.

### Clinical Requirements

The JACIE standard defines the clinical requirements for the compliance of BMT centers. The clinical requirements for CAR-T cells therapies are: i) donor selection and evaluation; ii) infusion of the IEC product; iii) recipient care; iiii) evaluation and training of the personnel dedicated to the above activities; iiiii) product management.

#### Collection Requirements

JACIE standard provides guidelines to perform the apheresis for the collection of lymphocytes. In particular, the following points are discussed:

- Donor selection and evaluation;
- Aphaeretic product collection;
- Aphaeretic product labeling;
- Aphaeretic product release to the laboratory dedicated to its manipulation;
- Apheretic product transport;
- Evaluation and training of the personnel.

#### Processing Requirements

According to the EC Directives, only a processing laboratory recognized as tissue establishment (TE) can release the product and coordinate the transfer to the GMP facility for CAR-T cell manufacturing. JACIE standard identifies the criteria and guidelines for: 1) the receipt of apheretic product; 2) final product (CAR-T product) labeling; 3) final product (CAR-T product) release; 4) final product (CAR-T product) transport; 5) evaluation and training of the personnel.

#### Advanced Therapeutic Medicinal Products Regulation

CAR-T cell therapies are included in the category of ATMPs within the definition of gene therapy medicinal product. According to the Committee for Advanced Therapy, the CAR-T Product is an ATMP product: “*products consisting of cells or tissues may scientifically be at the border between Tissues and Cells directive (Directive 2004/23/EC) and the ATMP regulation”*. The classification of an ATMP as a biological product determines a wider regulatory framework and the subsequent requirements for the development and the marketing authorization. These need to be considered in association with the specific framework for ATMPs, *Regulation 1394/2007/EC*, which was approved on December 30, 2008: “*ATMP are authorized centrally through the European Medicine Agency (EMA). They benefit from a single evaluation and authorization procedure. EMA continues to monitor the safety and efficacy of ATMPs following their approval and commercialization. This Agency also provides scientific support to the developers of these products in the design of pharmacovigilance and risk management systems to monitor the safety of these medicines*”. This regulation provides the overall framework on ATMPs for those products, which are intended to be placed in the market of EU Member States. In addition, *Directive 2009/120/EC* updated the definitions and detailed scientific and technical requirements for advanced therapies. ATMPs are regulated by both guidelines of medicinal products and that of medical devices. On the 25th of May 2017, two additional regulations on medical devices were identified (*European Commission, 2017a*). For the development of advanced therapies in EU, the development and approval of clinical trials are in charge of the individual national competent authorities. However, for marketing authorization, all ATMPs are evaluated through centralized procedures ensuring that they benefit from a single evaluation and authorization across EU. Two committees are responsible for the scientific evaluation and approval of ATMPs: the CAT and the Committee for Medicinal Products for Human Use (CHMP) (European Medicines Agency, 2018a). The CAT is the EMA committee responsible for classifying, assessing the quality, safety, efficacy of ATMP, in order to follow the scientific progress in the field and to provide an opinion on each ATMP application submitted to the EMA, supporting the final decision by the CHMP.

## The CAR-T CELL Process

The process for the manufacturing and infusion of CAR-T cells is relatively complex not only for the clinical aspects, but also from an organizational and regulatory point of view. The production and infusion of CAR-T cells require a specific organizational structure; the clinical unit task force includes physicians and nurses, and numbers of other clinical and healthcare personnel with long-term expertise in the care of patients with either hematological malignancies or solid tumors and in HSCT procedures. Therefore, it is important to have a detailed list of the personnel and multi-disciplinary expertise involved in this process and their role and relationship.

### Patient Evaluation and Selection

The clinical CAR-T unit is in charge of the evaluation and selection of patients eligible for CAR-T cell therapy. This represents a critical task since the demand of patients in need of such treatment will increase overtime while the availability of CAR-T cells are still limited—at least so far—to a relatively low number of clinical centers. Stringent eligibility criteria are required in order to select those patients that may benefit from CAR-T cell administration. The decision to treat a patient with CAR-T cell therapy should be made in the context of a multi-disciplinary team. The main factors that influence the suitability of a patient to CAR-T cell treatment are represented by the medical history, the clinical conditions, the disease burden, and life’s expectancy. In particular, disease burden at the time of evaluation represents a critical consideration, as patients with low tumor burden experience less treatment-related toxicities and have superior clinical responses (22). However, in real life, most patients have aggressive malignancies that need to be carefully managed along with the timeline and processes, such as insurance authorization, apheresis procedure, and manufacturing, to obtain the medicinal product for infusion. Moreover, clinicians should take into account the risk of disease progression and the related complications that could occur until the expected date of CAR-T cell infusion. Finally, also manufacturing failures should be foreseen, since in this case alternative therapies have to be considered.

### Lymphocyte Collection

The apheresis unit is in charge of the patient/donor suitability assessment for the procedure. The collection procedure follows the clinical protocol guidelines. The following steps involve the labeling of the apheresis product and the delivery to the cell processing laboratory.

### Lymphocyte Processing (Minimum Manipulation)

The TE may perform the manipulation to isolate and cryopreserve lymphocytes according to standardized and validated procedures. The cryopreserved lymphocytes are then delivered to the good manufacturing practice (GMP) facility that can be either part of a pharmaceutical company or an independent site. Alternatively, fresh collected lymphocytes may be sent directly from the TE to the GMP facility that will proceed to lymphocyte isolation and subsequent processing.

### GMP Production and Transport

The GMP facility is responsible for the manufacturing, control, and release of the drug products, according to ATMP regulation. The product is then released and shipped to the CAR-T unit. CAR-T cells are shipped in the form of frozen cells to the clinical center. The pharmacy unit will receive, register, and handover the cells to the TE for temporary storing. The final process involves cell thawing for the administration to the patient. This step may be performed either by the TE that will provide the cells to the clinical unit or directly by the clinical unit.

### Treatment of Patient Before the Infusion of CAR-T Cells

CAR-T cell infusion is administered after a brief course of chemotherapy. The aim of such chemotherapy is to control, at least in part, the disease during the period between lymphocyte collection and the final infusion of the CAR-T cell product (52). However, it is advisable that the treatment with CAR-T cells might be performed before the achievement of disease complete remission, since the target molecules of CAR-T cells might be no longer detectable. “Bridge” chemotherapy plays an important role in acute lymphoblastic leukemia (ALL) as leukemic blast burden at the time of CAR-T cell infusion correlates with an increased risk of cytokine release syndrome (CRS). The overall tumor burden and the involved sites are additional factors to be considered also in lymphoma patients: many regimens are indicated in this phase, and the choice must take into account the type of histology, the burden of disease, previous treatments, and the fragility of the patient. However, the “bridge” chemotherapy should be given only after leukapheresis in order not to affect the quality of CAR-T cell product (53). Two to seven days before CAR-T cell infusion, patients receive a lymphodepleting conditioning regimen to get rid of immune cells and factors endowed with immune suppressive properties and to enhance CAR-T cell functions and engraftment (54). The optimal conditioning regimen has not been defined yet, but the majority of studies employed a combination of fludarabine and cyclophosphamide. Experiences at the NCI (55) and Fred Hutchinson Cancer Center (56) demonstrated that aggressive lymphodepleting regimens are associated with the increase of both CAR-T cell expansion and toxicity. Of note, certain drugs such as check point inhibitors or other biological drugs (*i.e.*, alemtuzumab, daratumumab, and brentuximab vedotin) should be avoided since they may interfere with expansion and persistence of CAR-T cells after infusion (53). Infectious complications and organ toxicities should be minimized during bridging chemotherapy (57).

### CAR-T Cell Infusion

CAR-T cell infusion requires an appropriate clinical setting with dedicated gene therapy rooms, medical and nursing standard of practice protocols, documentation and verification procedures for administration. CAR-T cells’ targeted therapies frequently induce toxicities that can be mitigated by a planned hospital organization. Comprehensive training should be provided to all categories of personnel including scientists, nurses, and physicians, and close collaborations with a range of other specialists, especially intensive care unit (ICU) staff and the neurology/neuroimaging services, are required (58). Indeed, the management of patients undergoing CAR-T cells requires two necessary conditions: a structured clinical unit with well-established procedures to take in charge of patients developing acute immunological complications and intensive care unit (ICU) close to the clinical unit. Protocols for the interactions between the two units have to be established at each center.

### Post-Infusion Follow-Up

During the pre- and early post-infusion period, patients are generally admitted to inpatient unit, as the rapid *in vivo* proliferation of CAR-T cells may be associated with adverse events, such as CRS and neurotoxicity (59–61). Currently, rapid advances have been made in the identification of clinical and biological predictors and in the design of appropriate standards of care to mitigate the severity and long-term consequences of these complications (62). CRS is the most common side effect following CAR-T cell therapy, occurring in 30 to 100% of cases, with CRS of grades 3–4 in 10–30% of patients (63). CRS may present with a spectrum of manifestations from low-grade fever to fulminant multiorgan failure (64–66). In some studies, approximately 10–15% of CAR-T cell patients required vasopressor support for hypotension and/or mechanical ventilation (61, 67). The mainstays of treatment for severe CRS include the anti-IL6-R antibody (tocilizumab), anti-IL6 antibody (siltuximab), high-dose steroids, and supportive care delivered in ICU (59, 61). Patients treated with CAR-T cells may develop concomitantly with CRS or as an isolated feature, a potentially fatal neurological toxicity, including delirium, encephalopathy, nerve palsies, and seizures, whose mechanism seems to be related to systemic inflammation due to rapid CAR-T cell expansion (61, 64). Different products have variable rates and patterns of toxicity, and clinical judgement remains the best method to assess CRS. Tisagenlecleucel induced severe CRS (grade 3 or 4 by the UPenn toxicity criteria) in 48% of pediatric and young adult ALL patients in the ELIANA trial (21) and 28% of adult patients with diffuse large B cell lymphoma (DLBCL) in the JULIET trial (52), while the ZUMA-1 trial reported a 13% rate (by NCI Common Terminology Criteria for Adverse Events) for axicabtagene ciloleucel in adult DLBCL patients (23). Nurses and ancillary medical personnel should be adequately trained about the recognition of CAR-T cells’ specific care and toxicities in order to expedite the evaluation for the transfer to ICU and treatment with tocilizumab. The second most common adverse event is the immune effector cell-associated neurotoxicity syndrome (ICANS) (59). The symptoms and signs are non-specific, and the severity is correlated with the increase of specific biomarkers such as C-reactive protein, ferritin and IL-6 (59, 68). ICANS incidence has been reported in 12–55% of cases. Patients are monitored by nursing tools to identify early manifestations of CRS or neurotoxicity. In the latter case, serial cognitive testing and neurological expertise are needed. In addition to infectious events, CAR-T cell recipients are at increased risk of potential medium-term complications, depending on the type of CAR-T cell product, including delayed tumor lysis syndrome, delayed hemophagocytic lymphohistiocytosis/macrophage activation syndrome and CRS, B-cell aplasia, hypogammaglobulinemia. Neutropenia, thrombocytopenia, and anemia are commonly observed in CAR-T cell treated patients, but generally resolve over several months through the administration of growth factors following the observation at early stages of adverse events.

## The CAR-T Unit

In order to guarantee the processes illustrated above and the regulation compliance, it is necessary to establish a dedicated organization that comprises different units within the clinical center. The clinical unit has the responsibility to select the patient, to evaluate the donor, to perform the infusion of CAR-T cell product, and to manage the follow-up activities. The apheresis unit collects mononuclear cells and transfers them to TE. The TE performs (if required according to the specific protocol) the processing, labeling, and storage of the aphaeretic product and organizes the shipping to the GMP facilities. Under the responsibility of the pharmacy department, the TE receives the CAR-T product, performs the temporary storage, and transfers the product to the clinical unit for infusion. The pharmacy has the responsibility to receive the drug product and to delegate the TE to store the product. ICU supports the clinical unit in the management of the patients in case of adverse events, together with several medical support consultants.The Organizational Model of the CAR-T Unit

A CAR-T unit can be organized according to either a centralized or decentralized structure. A CAR-T center requires the work of a multi-disciplinary team.

**Figure 1:**
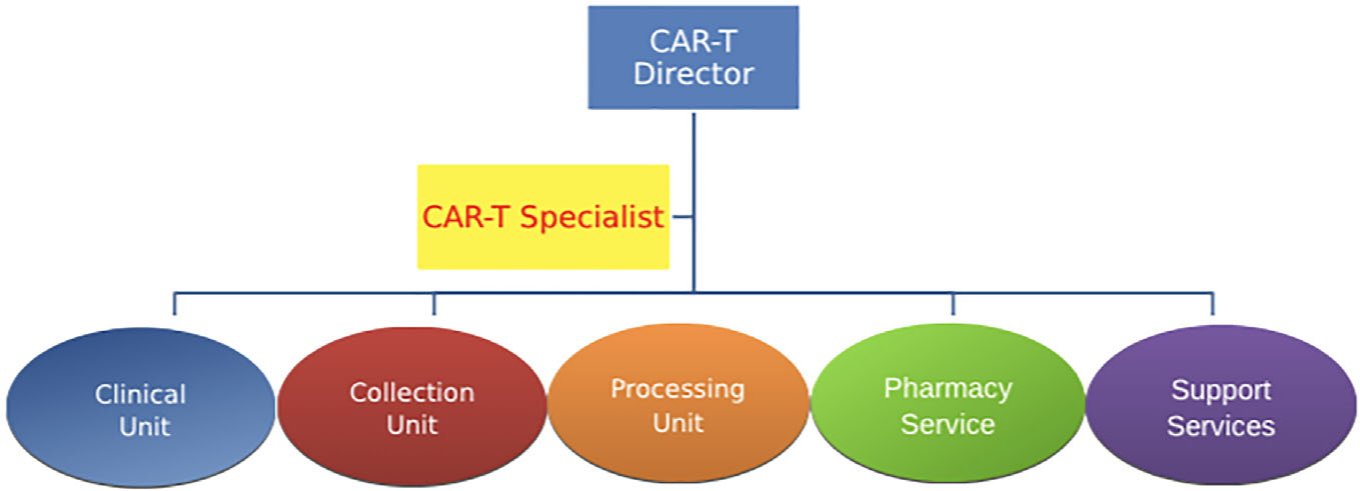
Centralized CAR-T unit This model is based on the centralization of the activities with a single unit, and it is useful for a single unit involved in CAR-T cell process. CAR-T specialist is a unique figure coordinating the whole process.

The centralized CAR-T unit is a model based on the centralization of the activities with a single unit. This structure is functional for the organizational entities where knowledge and different expertise of the staff are in a single unit. Usually in this organizational modality, the CAR-T unit corresponds to the HSCT Program (**Figure 1**). This model is useful where there is a single Unit of Hematology or Onco-hematology.

**Figure 2:**
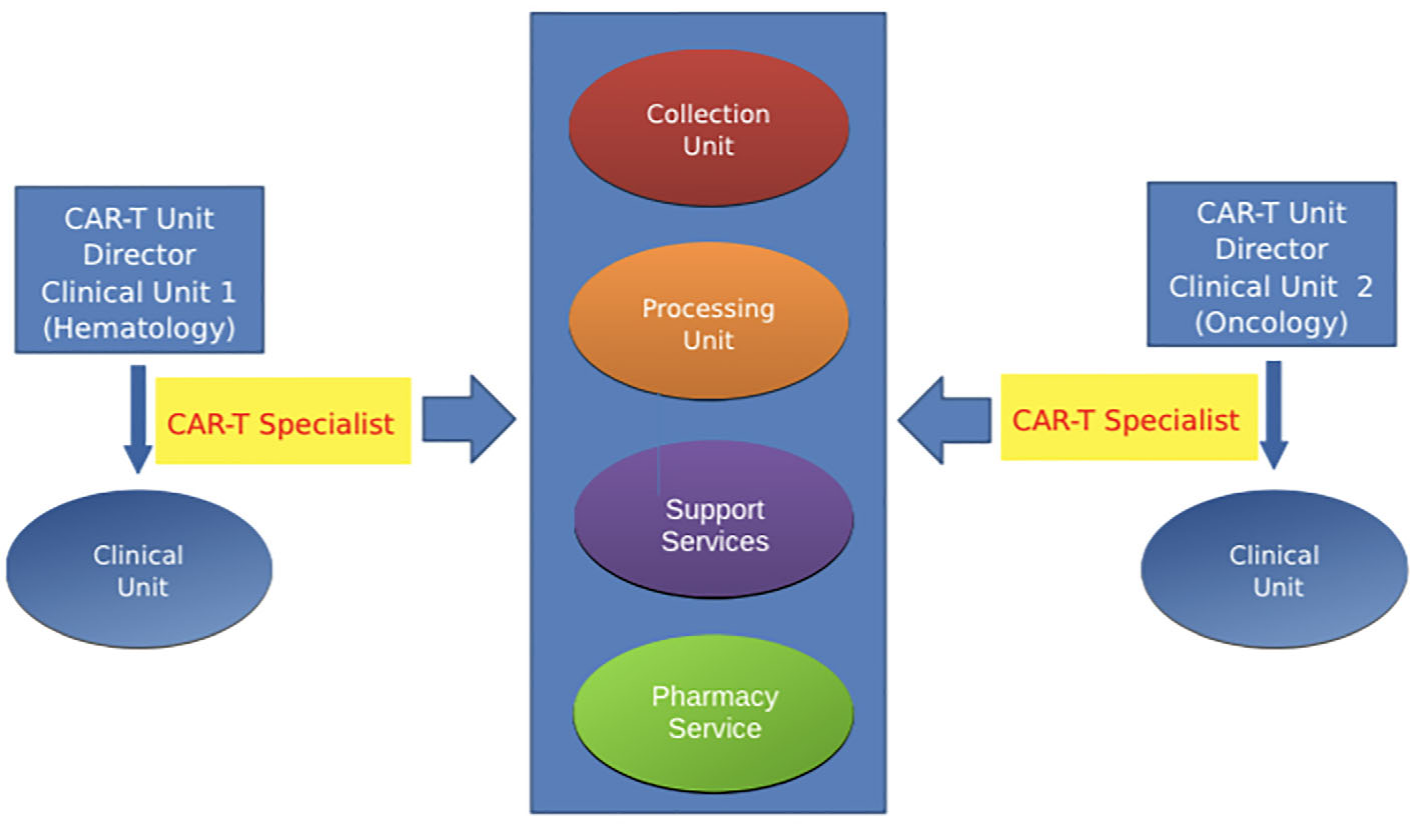
Decentralized CAR-T unit This model is suitable when cell therapies occur under the responsibility of different subjects (complex organizational structures with separate business unit for Oncology and Hematology). CAR-T specialist is present in each involved unit.

The second model, the decentralized CAR-T Unit, is a model suitable for entities where the infusion of cell therapies occurs under the responsibility of different subjects. This model is implemented in complex organizational structures in which the types of IECs utilized for cell therapy are different and are administered to patients with either blood malignancies or solid tumors (**Figure 2**). This model is useful in Hospitals and Institutes with separate business units for Oncology and Hematology even within a single CAR-T center. This is useful for the current different values of Oncology and Hematology and the continuous evolution of their relationship. Furthermore, this model allows having a CAR-T unit even in the context of subdomain of Oncology, such as Neuroncology.

## The CAR-T Specialist

The CAR-T specialist represents a critical profile in the multi-disciplinary and multi-professional team of the CAR-T unit. This profile is dedicated to the compliance with regulatory and organizational requirements for ATMP therapies. Therefore, the CAR-T specialist is a key person within the CAR-T Unit with knowledge of the scientific principles of CAR-T cells, regulatory and quality requirements, management and administrative skills, organizational knowledge and experience in clinical trial. The CAR-T Unit Director or Transplant Program Director can identify one person on the team (Centralized) or more than one (Decentralized) with responsibility for the organization and quality system of the CAR-T unit. The CAR-T specialist can be represented by the staff responsible for managing the quality system of the BMT program but with documented experience in the aforementioned topics.

### Education and Knowledge of CAR-T Specialist

The requirements of the quality manager for BMT centers are detailed in the JACIE Standard, while for the CAR-T specialist those requirements need to be extrapolated and implemented. In particular, the CAR-T specialist has scientific knowledge of CAR-T cells and cell-based therapies, and in addition to the skills listed above, a minimum of 10 h of training and educational activities related to the IEC and the management of quality is mandatory. This role can be covered by a person with the following requirements:

- Scientific degree (physician, biologist or pharmacist);
- Experience of 5 years in the field of stem transplant center or in a processing laboratory or in apheresis or oncology unit;
- Good knowledge of the quality requirements in a tissue establishment, transplant centers (*i.e.* JACIE-FACT) or blood centers;
- Good knowledge of the leukapheresis process;
- Good knowledge of the national requirements of the tissue and blood regulations.

### The Job Description of CAR-T Specialist

CAR-T specialist bears the responsibility for the organizational management of the CAR-T unit. The following are the major tasks:

- Coordinating the organizational activities of the CAR-T center;
- Developing the organization system;
- Managing quality system;
- Managing personnel training and education;
- Creating a project plan for the management of the CAR-T cell therapies;
- Planning and managing audits for the CAR-T center;
- Collecting data and indicators;
- Performing training related to the procedures for managing the CAR-T cell therapies;
- Coordinating the facilities’ activities in the CAR-T program.

The important role of the CAR-T specialist is to coordinate the activities with the different units involved in the CAR-T process. Particularly, the following will be involved:

*- Transplant Program (clinical unit, collection unit and processing unit):* the CAR-T specialist will manage the relationship between the facilities involved in the transplant program according to the CAR-T requirements and JACIE requirements;
*- Pharmaceutical companies:* the CAR-T specialist represents the bridge between the pharmaceutical company and the GMP facility. The CAR-T specialist maintains the relationship with the pharmaceutical company and GMP facility for sponsored studies or for commercial usage of cell-based therapies. Moreover, this person will supervise the education and training activities of members of the teams;
*- Other internal facilities:* the CAR-T specialist guarantees that the team members involved in the activities of the CAR-T unit have the necessary knowledge of their specific tasks. In addition, the CAR-T specialist coordinates with the pharmacy, the clinical trial office, and the ICU.

The CAR-T specialists are responsible for developing and managing the quality system of the CAR-T Unit according to: i) national regulations; ii) requirements of the GMP Facility; iii) requirements of the FACT-JACIE Standards.

Moreover, the CAR-T specialist is in charge of coordinating the preparation of documents for the registration of the activities of the facilities, to maintain the quality system, and to perform internal audits in the CAR-T Unit.

Because the CAR-T unit is incorporated in several facilities, the CAR-T specialist should be in charge of a multi-disciplinary and/or multi-professional group composed of:

- Quality manager of the Transplant Program
- Quality Manager of the Apheresis unit
- Quality Manager of the processing unit
- Quality Manager or Qualify Person of the GMP Facility (if present)

According to the specific organization, the role of the CAR-T specialist could be managed by the Transplant Program Quality Manager. The role and responsibilities of the CAR-T specialist will vary for the two models: in the first model, the CAR-T specialist will manage all the activities of the team(s), while in the second model each facility will have a CAR-T specialist (one for the oncology clinical unit, one for the hematology unit, and one for the processing and collection unity). A plan of coordination and collaboration between these CAR-T specialists will be required.

## Conclusions

The manufacturing and management of CAR-T cells comprise complex processes, various facilities, including clinical, processing and apheresis units, intensive care unit, pharmacy, medical consultants, GMP facilities, and pharmaceutical companies. Moreover, the CAR-T process has to be compliant, at European level, with different regulations, including tissue and cells, blood and ATMP legislation. According to the complexity of the process and the multi-disciplinary implications, the role of the CAR-T specialist becomes critical and indispensable. This expert figure guarantees that all the requirements are applied along the flow and processes within the CAR-T Unit, including the transfer of knowledge, the interconnections among multiple facilities, and the coordination and management of all the organizational aspects. The scientific and clinical developments of CAR-T cells are under rapid evolution and require specialized and long-term expertise personnel, accurate connections, and coordination of multiple facilities to make such living drugs available to a broad number of cancer patients.

## Author Contributions

All authors contributed to the article and approved the submitted version.

## Conflict of Interest

VI is the CEO and founder of ICMED S.r.L.

The remaining authors declare that the research was conducted in the absence of any commercial or financial relationships that could be construed as a potential conflict of interest.
